# TiO_2_ nanoparticles functionalized by Pd nanoparticles for gas-sensing application with enhanced butane response performances

**DOI:** 10.1038/s41598-017-08074-y

**Published:** 2017-08-09

**Authors:** Nan Chen, Dongyang Deng, Yuxiu Li, Xu Liu, Xinxin Xing, Xuechun Xiao, Yude Wang

**Affiliations:** 1 Department of Physics, Yunnan University, 650091 Kunming, People’s Republic of China; 2 School of Materials Science and Engineering, Yunnan University, 650091 Kunming, People’s Republic of China; 3 International Joint Centre for National Optoelectronic Energy Materials, Yunnan University, Kunming, 650091 People’s Republic of China

## Abstract

Pd functionalized TiO_2_ nanoparticles were synthesized by a facile hydrothermal method. The structure, morphology, surface chemical states and surface area were characterized by X-ray diffraction (XRD), transmission electron microscopy (TEM), X-ray photoelectron spectroscopy (XPS), and N_2_ adsorption-desorption isotherms, respectively. The as-synthesized pure and Pd functionalized TiO_2_ nanoparticles were used to fabricate indirect-heating gas sensor, and the gas-sensing characteristics towards butane were investigated. At the optimum temperature, the sensors possess good response, selectivity, response/recovery, repeatability as well as long-term stability. Especially for the high response, the response of 7.5 mol% Pd functionalized TiO_2_ nanoparticles based sensor reaches 33.93 towards 3000 ppm butane, which is about 9 times higher than that of pure TiO_2_ nanoparticles. The response and recovery time are 13 and 8 s, respectively. Those values demonstrate the potential of using as-synthesized Pd functionalized TiO_2_ nanoparticles as butane gas detection, particularly in the dynamic monitoring. Apart from these, a possible mechanism related to the enhanced sensing performance is also investigated.

## Introduction

Butane, a colorless, slight irritant and easily liquefied gas, which is widely used in outdoor cooking, subcritical extraction^1, 2^, refrigerant^3, 4^ and the raw material of organic synthesis^5^. However, butane has a flash point of −60 °C and an explosion limit of 1.5 ~ 8.5%. This means that it is easy to blast, and has a threat to the property and life safety of people. When the butane concentration reaches 1000 ppm in air, the phenomenon of dizziness, drowsiness and drunk, even coma, will appear^6^. In addition, the toxic and noxious gases of carbon monoxide and carbon dioxide produced from butane combustion are a hidden danger to human body health. Hence, the detection of the butane is necessary.

Up to now, the sensing materials, such as MFe_2_O_4_ (M = Zn, Cu, Ni, and Co)^7^, SnO_2_^8^, ZnSnO_3_^9^, ZnO^10^, Fe_2_O_3_^11^, ZnCo_2_O_4_^12^ were used to detect butane. However, the responses of the articles show the lower value. For example, E.R. Kumar reported that the response of Mn substituted ZnFe_2_O_4_ for 1000 ppm butane is 2^7^. It is reported that to 500 ppm butane, the sensor response of the hollow ZnSnO_3_ microspheres is 5.79 at the optimum operating temperature of 380 °C^9^. The maximum response of the sensor based on γ-Fe_2_O_3_ to 1000 ppm n-butane at 300 °C reported by I. Ray *et al*. is about 10^11^. For ZnCo_2_O_4_ spinel synthesized by S. Vijayanand exhibits high response (~72 towards 50 ppm butane), but the response time (~85–90 s) and recovery time (~75–80 s) are a little longer, which perhaps cannot satisfy the timely monitoring needs^12^. In this case, searching for a potential butane-sensing material which has higher response and shorter response/recovery time is deserved.

TiO_2_, a wide energy gap semiconductor, has been intensively studied as a key material for fundamental research and technological applications in the fields of semiconductors, lithium-ion batteries^13^, photocatalytic decomposition^14^ and solar cell^15^, because of its good chemical stability, non-toxicity, abundance and low cost^16^. Its versatility makes it a promising use in sensing performance. However, TiO_2_ is a high resistance n-type semiconductor with conductivity that is too poor to be considered for gas sensing oxidative gases, and its low electrical conductivity inhibits its practical implementation as a gas sensor^17^. From the reported literatures, we realized that doping or adding precious metal is an effective strategy for enhancing the sensing performance. For instance, in C.L. Xiang’s work, well-dispersed Pd nanoparticles were decorated on the highly ordered TiO_2_ nanotubes by chemical reduction while TiO_2_ nanotubes were prepared through an anodic oxidation in an ethylene glycol aqueous solution. The results showed hydrogen sensor based on Pd nanoparticles decoration with the TiO_2_ nanotubes, while Pd nanoparticles can adsorb hydrogen to trigger the formation of Pd hydride, both high sensitivity and excellent selectivity have been achieved with rapid response and recovery^18^. E. Şennik *et al*. prepared TiO_2_ nanowires on Ti foil by hydrothermal method, obtained spider-web like structures were obtained, and these TiO_2_ nanowires were loaded with Pd.The experimental study reported that Pd-loaded TiO_2_ sensor shows enhanced response to 5000 ppm ethanol with 400 times better sensitivity compared to the pristine TiO_2_ sensor^19^. Pd loaded TiO_2_ nanorods (Pd-TiO_2_ NRs) in E. Şennik’s work were synthesized via the chemical vapor deposition (CVD) process. The result showed that Pd-loaded TiO_2_ can be used as a sensing material for improving sensor abilities, the reason for using Pd is the higher catalytic activity of Pd particles on O_2_ adsorption and desorption^20^. These results reveal that doping or adding precious metal is an effective measure to enhance the gas sensing performance of TiO_2_ nano-materials. In the meantime, to the best of our knowledge, there are fewer reports on the butane sensing properties of Pd functionalized TiO_2_ nanoparticles, which were prepared by hydrothermal method.

In this paper, Pd functionalized TiO_2_ nanoparticles were obtained by a simple, facial hydrothermal method. The Pd functionalized TiO_2_ nanoparticles were then used as sensing material for indirect heating sensor. The gas-sensing performances of the sensors were measured, showing good sensing properties towards butane, particularly with the high response and fast response/recovery time. In addition, the sensing enhancement mechanism of the materials is discussed.

## Results and Discussion

**Figure 1:**
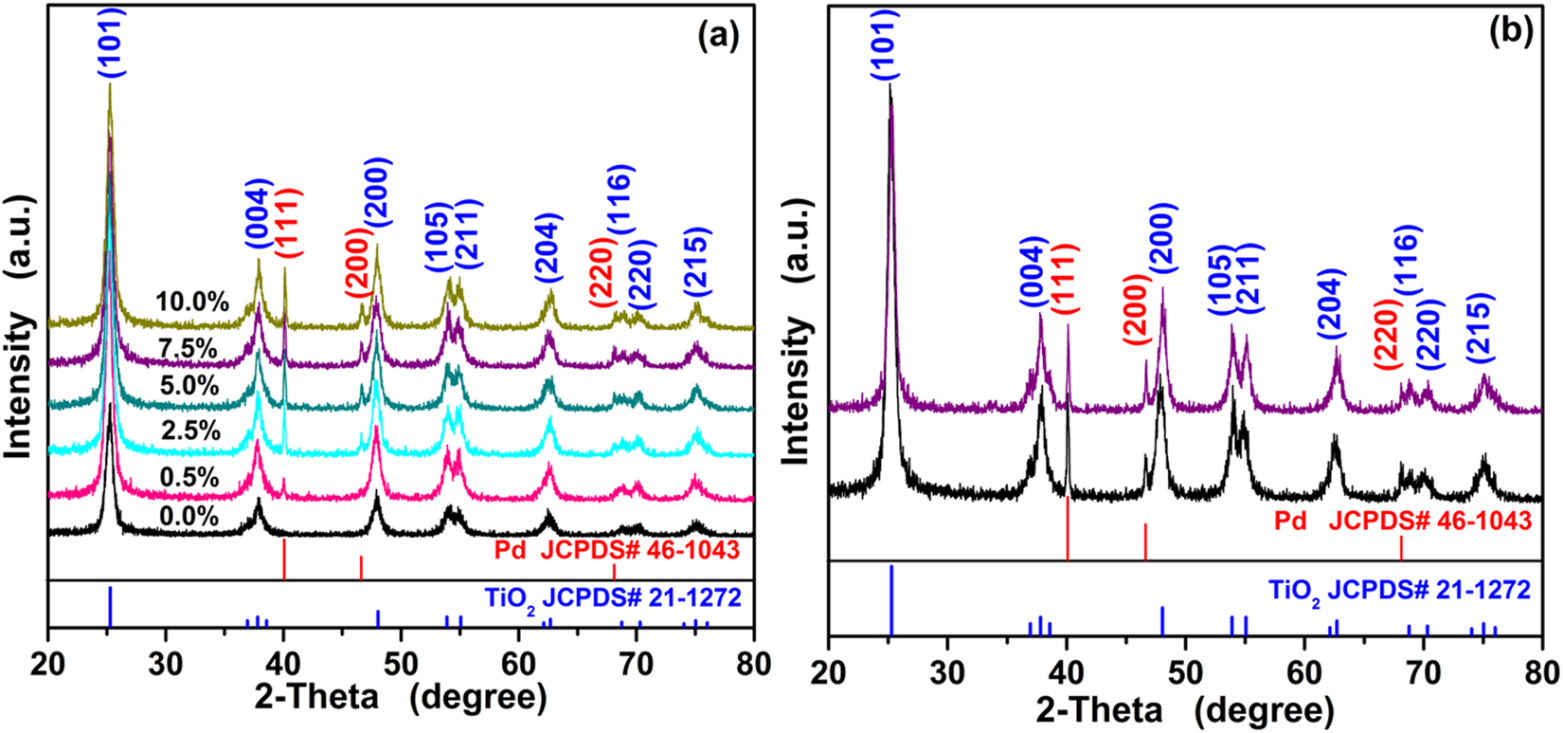
(**a**) The XRD patterns for as-synthesized pure and Pd functionalized TiO_2_ nanoparticles; (**b**) XRD patterns for as-synthesized original PTO-4 nanoparticles (the black) and PTO-4 nanoparticles after calcination (the purple) at 400 °C for 1 hour.

The structural features of the as-synthesized Pd functionalized TiO_2_ nanoparticles were analyzed by XRD. As shown in Fig. 1(a), one can find that all the experimental diffraction peaks can be perfectly indexed to TiO_2_ (anatase, JCPDS No. 21-1272, space group: *I4*_*1*_ */amd* (141)) and Pd (JCPDS No.46-1043, space group: *Fm-3m* (225)). No obvious peaks from impurity are observed, indicating the high purity of the obtained products. The broader diffraction peaks, for instance, the overlapping of (103), (004) and (112), suggest the small crystallite size of the products. Besides, the appearance of Pd diffraction peaks indicated that Pd could be incorporated on the surface of TiO_2_ instead of doped into lattice of TiO_2_ successfully.

Moreover, in the process of fabricating sensors, we mentioned that the thickness of the sensitive body was dried and calcined in air at 400 °C for 1 h. Hence, the structural features of the sample after calcination were also analyzed by XRD, which was shown in Fig. 1(b). One can see that the experimental data are in agreement with anatase TiO_2_ (JCPDS No. 21-1272) and Pd (JCPDS No. 46-1043), indicating that the structural features have no changes before and after calcination.

**Figure 2:**
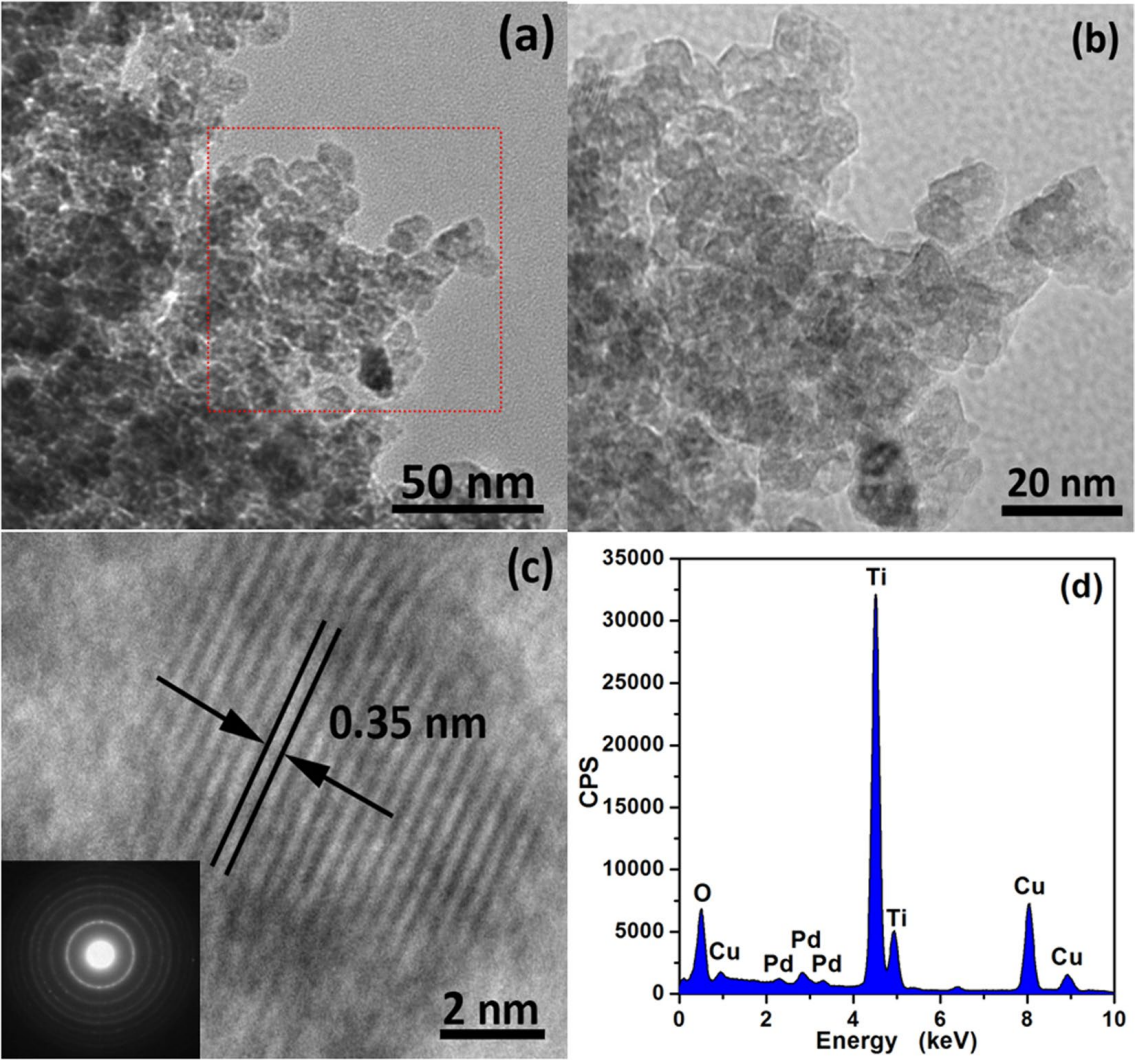
TEM image and magnified TEM image of as-synthesized products, (**a**) TEM image of PTO-4 nanoparticles; (**b**) magnified TEM image of PTO-4 nanoparticles; (**c**) the corresponding HRTEM image with obvious TiO_2_ lattice fringes (the inset is the selected area electron diffraction pattern), and (**d**) the corresponding spectrum of EDX.

The morphologies of as-synthesized Pd functionalized TiO_2_ nanoparticles were performed by TEM. Figure 2 shows the structural features of PTO-4 (7.5 mol% Pd functionalized TiO_2_ nanoparticles). As shown in Fig. 2(a), one can observe that the as-synthesized products have a rather uniform analogous shape as well as size. Figure 2(b) shows the magnified area of Fig. 2(a), one can see that the pore structure existed on the surface of PTO-4 nanoparticles, and the sizes of PTO-4 nanoparticles are in the range of 10 to 15 nm. The high-resolution TEM of PTO-4 is shown in Fig. 2(c). The sets of lattice fringes with an interplanar distance of about 0.352 nm can be associated with the (101) lattice planes of anatase TiO_2_, the inset is the selected area electron diffraction pattern. However, no lattice fringes of Pd phases are found by elaborating examination of many HRTEM micrographs of PTO-4. The probable reason of this phenomenon can be attributed to the local concentration of Pd nanoparticles. To demonstrate the existence of Pd, composition analysis was examined using energy-dispersive x-ray spectrometry (EDX) as for PTO-4, as indicated in Fig. 2(d). The peaks of O, Ti and Pd (Cu peaks are attributed to the copper grids) can be clearly seen, suggesting the high purity of the products.

**Figure 3:**
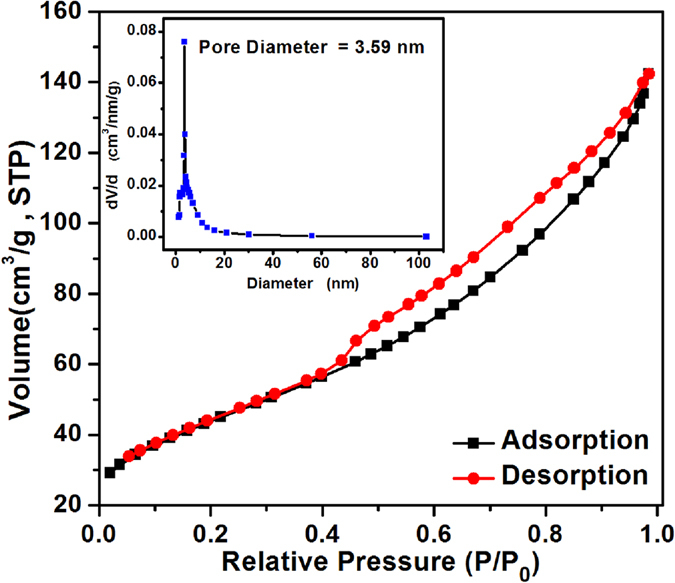
N_2_ adsorption-desorption isotherms of as-synthesized PTO-4 nanoparticles, and the inset is the corresponding pore size distributions.

Nitrogen adsorption-desorption isotherms measurements were performed to detect the surface adsorption properties of the sample. The N_2_ adsorption-desorption isotherms are shown in Fig. 3. The isotherm can be categorized as type IV with small hysteresis loops observed at a relative pressure of 0.4 to 0.95. The surface area estimated from the BET method is 155.51 m^2^ g^−1^. Inset of Fig. 3 illustrates its corresponding pore size distributions obtained from desorption branches. It can be concluded from the pore size distributions that PTO-4 has pores with diameter of about 3.59 nm, which is in good agreement with the TEM images of the sample. In virtue of the porous structure and high surface area, the PTO-4 nanoparticles will provide more active sites to get in touch with butane gas.

**Figure 4:**
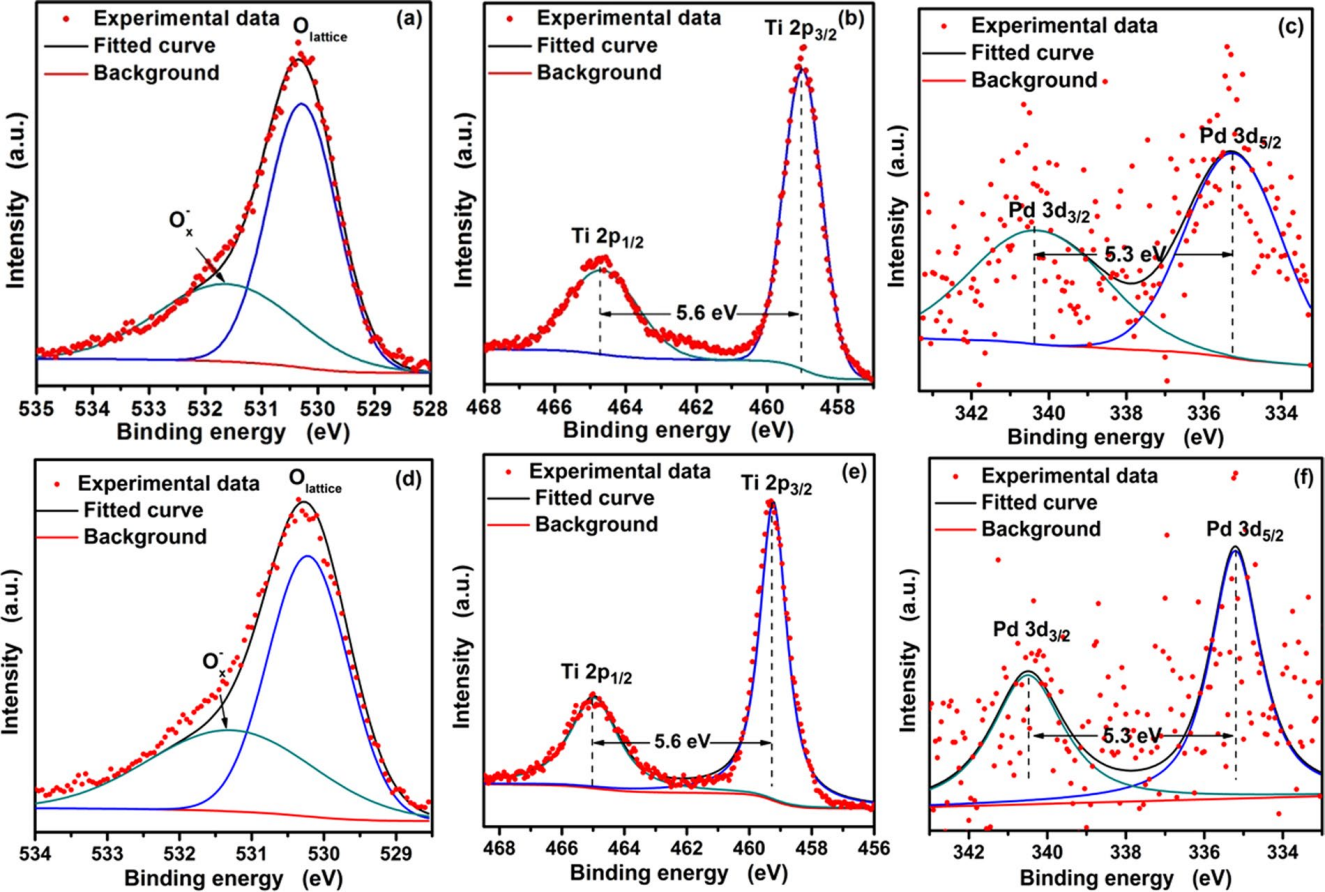
High-resolution XPS spectrum for PTO-4 nanoparticles: (**a**) O 1 s, (**b**) Ti 2p, and (**c**) Pd 3d; High-resolution XPS spectrum for PTO-4 nanoparticles after long-time response: (**d**) O 1 s, (**e**) Ti 2p, and (**f**) Pd 3d.

The surface/near surface chemical compositions of PTO-4 and PTO-4 after long-time response were analyzed by high-resolution XPS, as shown in Fig. 4. The O 1 s XPS spectra of PTO-4 are shown in Fig. 4(a). One can find that there are two kinds of oxygen in the surface: the peak center at 530.3 eV indexes lattice oxygen (O_lattice_) while the peak at 531.6 eV presents adsorbed oxygen $({{\rm{O}}}_{{x}}^{-})$ ^21^. O_lattice_ is attributed to the oxygen ions in the crystal lattice, which is thought to be pretty stable and has no contribution to the gas response, meanwhile, ${{\rm{O}}}_{x}^{-}$ is attributed to the chemical absorbed oxygen ions, which has a very important role in the gas sensing property^22^. In Fig. 4(b), Ti 2p spectra of PTO-4 reveal the existence of two peaks of Ti 2p_1/2_ and Ti 2p_3/2_ at the position of 464.7 eV and 459.1 eV, respectively. The distance between these two peaks is equal to 5.6 eV, which is in good agreement with the energy reported for TiO_2_: the values correspond to the 2p binding energy of Ti (IV) ions^2^. Figure 4(c) shows the Pd 3d spectra of PTO-4, the splitting energy between the two peaks of Pd 3d_3/2_ and Pd 3d_5/2_ at 340.7 eV and 335.4 eV is equal to 5.3 eV, which can be attributed to metallic palladium (Pd^0^)^23, 24^. However, in the butane performance testing process, PTO-4 nanoparticles will be tested for a long time. So the high-resolution XPS spectra of PTO-4 after long-time response should be analyzed. The results are shown in Fig. 4(d–f). In Fig. 4(d), one can find that there are two kinds of oxygen on the surface of PTO-4 after long-time response. The splitting energy between Ti 2p_1/2_ and Ti 2p_3/2_ in Fig. 4(e) is 5.6 eV, indicating the existence of Ti^4+^; the splitting energy between Pd 3d_3/2_ and Pd 3d_5/2_ in Fig. 4(f) is 5.3 eV, indicating the existence of metal Pd. Those results show that the status of PTO-4 nanoparticles do not changed after long-time response.

**Figure 5:**
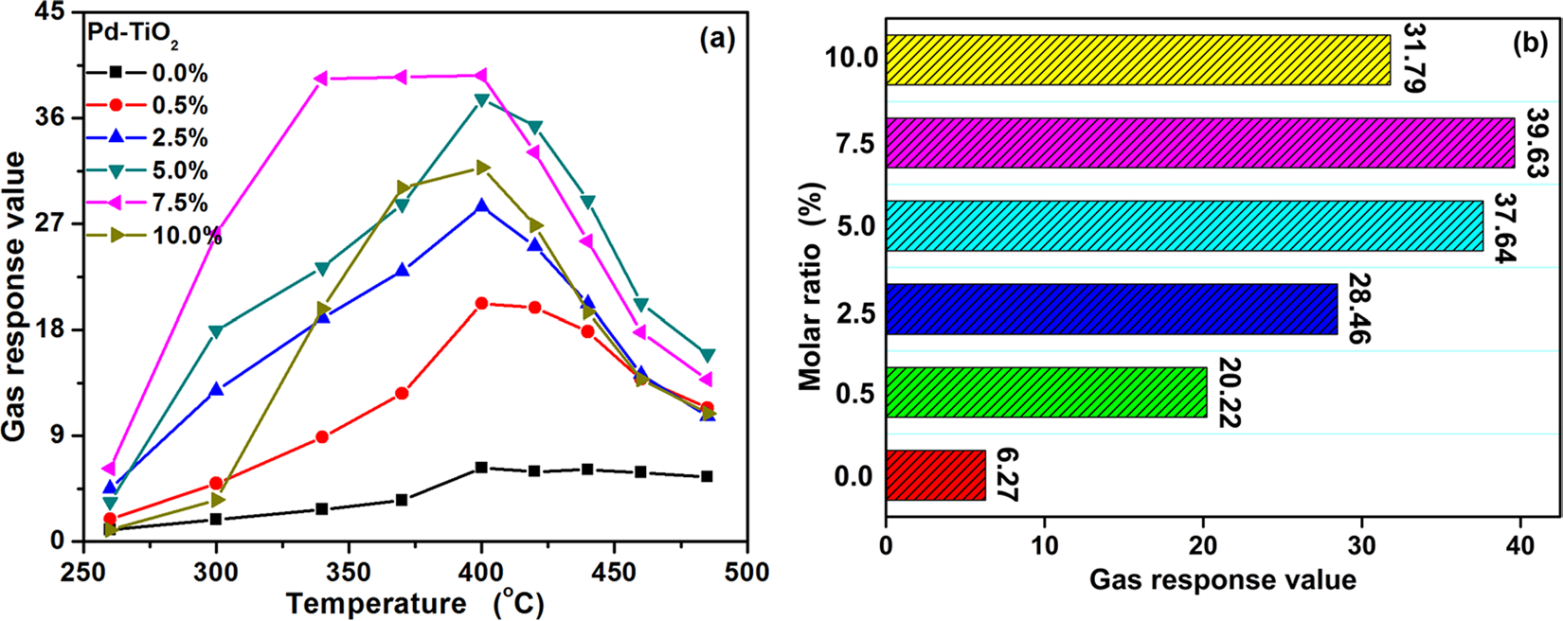
Gas responses of the sensors based on pure and different molar ratios of Pd functionalized TiO_2_ nanoparticles at different operating temperature toward 3000 ppm butane, and (**b**) the butane response of different mole percentage of Pd functionalized TiO_2_ nanoparticles fabricated sensors at the optimum temperature of 400 °C.

To evaluate the potential applicability of the fabricated gas sensor for butane detection, some fundamental gas sensing parameters were investigated. The operating temperature is one of the most important factors for gas sensor, which highly determines the nature of the sensing materials and the gas-sensing process between the gas and the surface of materials. Normally, the first approach is to confirm the optimum temperature of gas sensor. The sensitivity of the sensor fabricated with Pd functionalized TiO_2_ nanoparticles were analyzed at working temperatures ranging from 260 °C to 485 °C at butane ambient with a concentration of 3000 ppm. As shown in Fig. 5(a), one can obviously see that the gas response value of the Pd functionalized TiO_2_ nanoparticles based sensor first increases and then decreases with the temperature increasing. This case can be explained as follows. Obviously, at a low operating temperature, the low response (*β* = *R*_a_/*R*_g_) can be obtained for butane gas do not have enough thermal energy to react with the surface electron of the Pd functionalized TiO_2_ nanoparticles, which leads to a low response. With the operating temperature increasing, the thermal energy obtained is high enough to overcome the activation energy of the surface reaction^25, 26^. Moreover, when the temperature is higher, the lower gas adsorption ability of butane molecule causes the low utilization rate of the sensing material, leading to the reduction in response value^27, 28^. What’s more, among these sensors based on Pd functionalized TiO_2_ nanoparticles, PTO-4 exhibits the best response value towards butane. The response values of PTO-4 towards 3000 ppm butane at 340, 370 and 400 °C are 39.37, 39.5 and 39.63, respectively. The optimum temperature of PTO-4 is 400 °C. It’s worth noting that the response values of PTO-4 from 340 to 400 °C are almost equal. Thus, the relatively low temperature of 340 °C was chosen as the optimum temperature to the sensor based on PTO-4. The response of different molar ratio of Pd functionalized TiO_2_ nanoparticles at 400 °C are shown in Fig. 5(b). The response increased as the molar ratio of Pd was increased up to 7.5%, and the higher was little effective for increasing the response, which could be explained by a diffusional limitation of detection gas, i.e. the number of the reducing gas molecules are deficient compared with the number of the active mental sites; the excessive surface reaction activity caused by over Pd functionalized decreased the utility factors of the sensing body^29, 30^. Therefore, PTO-4 was chosen to further investigate the sensing properties.

**Figure 6:**
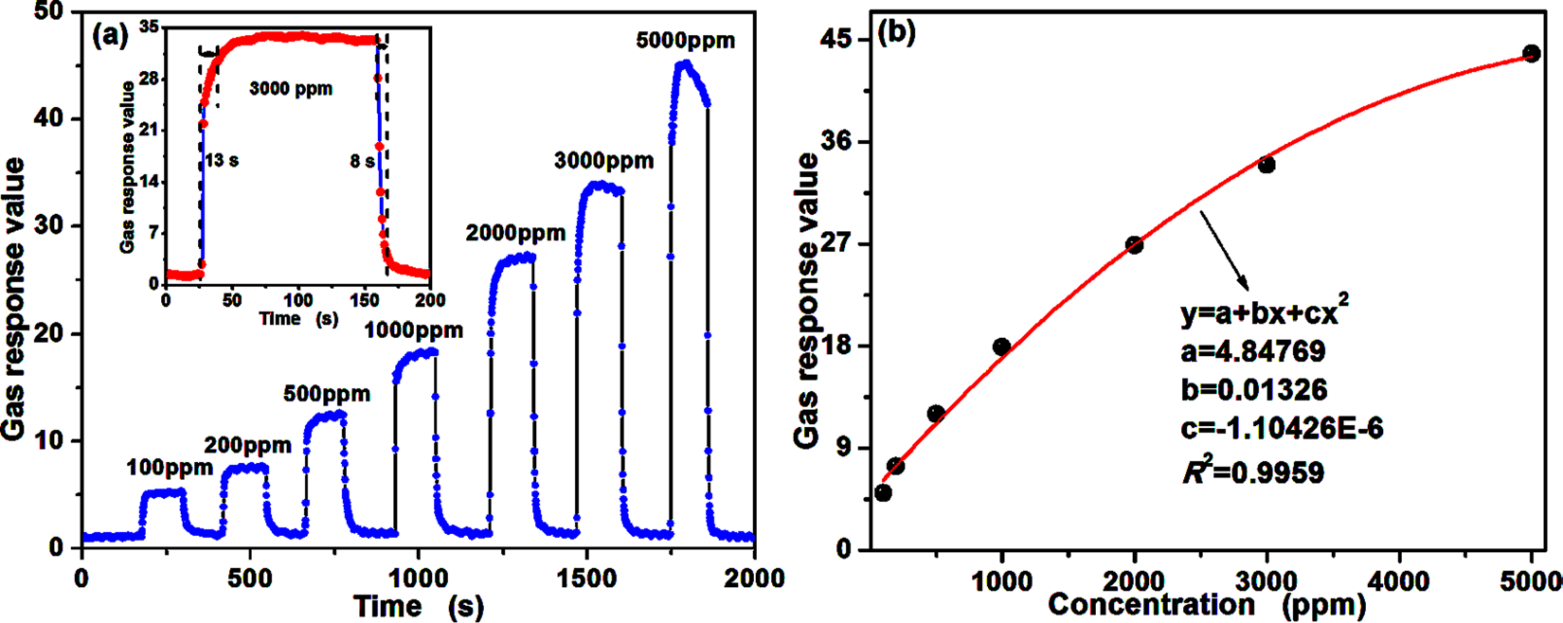
The butane gas-sensing properties of the sensor fabricated from PTO-4 nanoparticles at an operating temperature of 340 °C; (**a**) The dynamic response from 100 to 5000 ppm (the inset is the response/recovery time toward 3000 ppm butane gas), and (**b**) variation of gas response to different butane concentrations from 100 to 5000 ppm.

Therewith, Fig. 6(a) exhibits the dynamic response-recovery curve of PTO-4 towards different butane concentrations from 100 to 5000 ppm. Obviously, a good response-recovery property can be observed. The gas response keeps almost constant with only small fluctuations when it reaches its dynamic balance in both air and butane; the response value of the sensor increases along with the increasing concentration of butane. Meanwhile, the gas response also rapidly increases and decreases in response and recovery situations, respectively. That the response and recovery curve towards 3000 ppm butane show in the inset of Fig. 6(a) are to accurate understanding on the response-recovery property. The response and recovery times were calculated to be 13 and 8 s, respectively. In practical application, a good dependence of gas response on concentration benefits the quantitative measurement of the gas concentration.

The calibration curve is shown in Fig. 6(b) and experimental data from 100 to 5000 ppm were fitted as:

1 $$\beta =4.84769+0.01326{{\rm{C}}}_{{\rm{gas}}}-1{\rm{.}}10426\times 1{0}^{-6}{{\rm{C}}}_{{\rm{gas}}}^{{\rm{2}}}$$

where *β* is the gas response value, *C*_gas_ is the gas concentration. The correlative coefficient *R* ^2^ is 0.9959, indicating that the experimental data have a good agreement with the calibration curves. According to the equation (1), responses calculated towards 4000 ppm butane is 40.22. It is similar with the response of 5000 ppm butane (*β* = 43.76). The butane responses towards high concentration are similar and just have smaller increase, demonstrating that the sensor based on PTO-4 tends to be saturated when the butane concentration is over 4000 ppm.

**Figure 7:**
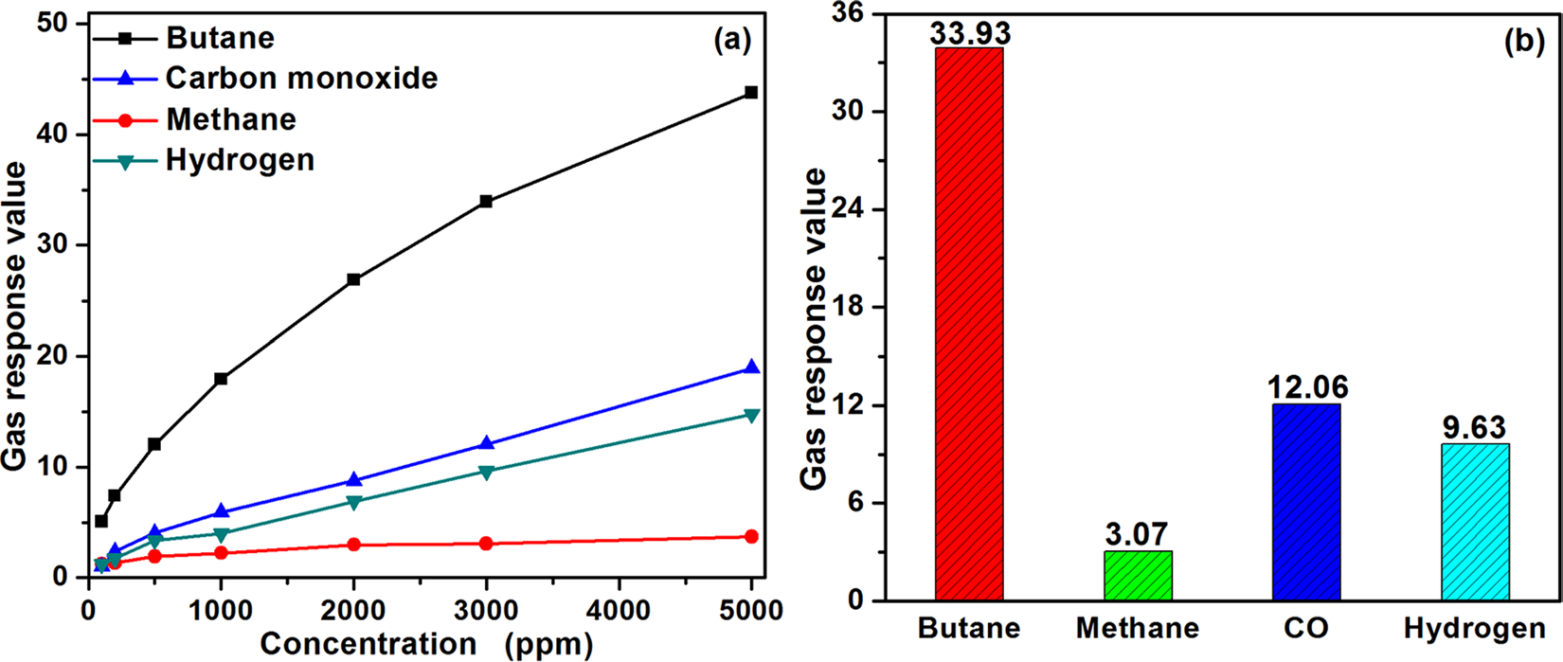
(**a**) Variation in gas response of the sensor based on PTO-4 nanoparticles to different tested gases from 100 to 5000 ppm, and (**b**) Gas response of PTO-4 based sensor towards 3000 ppm tested gases at the operating temperature of 340 °C.

What’s more, the gas response of common flammable gases, including methane, carbon monoxide and hydrogen were tested, the results are summarized in Fig. 7(a). One can see the response towards butane is the highest among the others. Figure 7(b) shows the response towards 3000 ppm butane is 33.93, and is about 11, 3 and 4 times higher than that of methane, carbon monoxide and hydrogen, meaning the sensor based on PTO-4 processes a good selectivity to butane. That the selectivity is closely related to the different reaction activities of the analyte gases to the sensing material is well known^31^. Contact with what we have explained above. We can find that the reaction activities of the gases can be obviously influenced by the operating temperature. Meanwhile, the Pd/Ti molar ratio can induce the increase of utility factors of the sensing body and thus change the surface reaction activity of the sensing material. Therefore, the selective detection of butane using PTO-4 can be realized via the synergistic control of the operating temperature and the molar ratio of Pd functionalization^25–29^.

**Figure 8:**
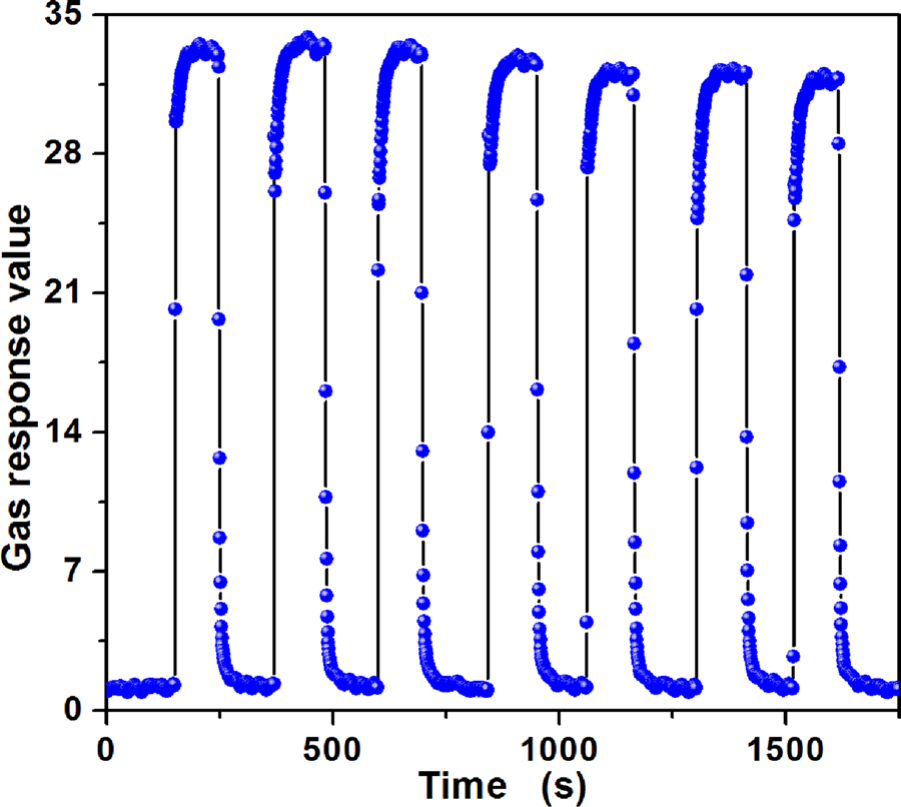
Repeatability of PTO-4 nanoparticles gas sensor to 3000 ppm butane at 340 °C. Response changes with time in continuous seven test cycles indicates the repeatability.

**Figure 9:**
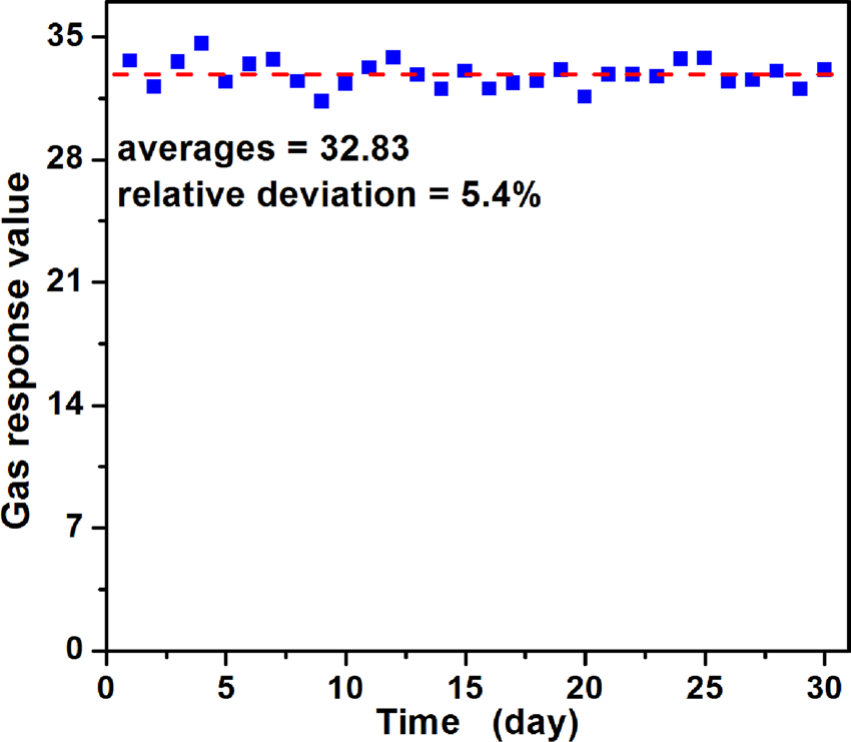
Long-term stability of PTO-4 nanoparticles gas sensor to 3000 ppm butane at 340 °C.

In addition, repeatability is also one important parameter which can be used to evaluate the reliability of a fabricated sensor. The repeatability of the sensor was investigated by testing 3000 ppm butane at 340 °C for seven cycles and the dynamic curve is shown in Fig. 8. The average response value is 32.59 with a small relative deviation of 2.27%, revealing that the sensor maintains its initial response amplitude without a clear decrease for seven successive sensing tests towards 3000 ppm butane. Moreover, in practical applications, the long-term stability of a gas sensor has attained much attention, for which the reliability of gas sensors and the service length were determined. To verify the stability of the sensor, the gas responses towards 3000 ppm butane over 30 days were tested at its optimum temperature. From Fig. 9, one can discover that the response value fluctuated around its average value of 32.83, and the relative deviation is below 5.4%, illustrating a good long-term stability of the gas sensor.

A brief summary of the sensing performances of various reported butane gas sensors are shown in Table 1. As it has been introduced, the responses of those butane-sensing materials are poor^21, 32–43^. Moreover, some of the reported sensors show a bad sensing performance or response/recovery property. For example, the sensor fabricated from 5% W-doped TiO_2_ possesses a response of 22.18 towards 5000 ppm butane at 420 °C^21^. B.K Min *et al*.^32^ reported the response for Pt-covered SnO_2_ thin film towards 5000 ppm butane is 4.6. The response of MgFe_2_O_4_ towards 2000 ppm butane is 3.45, while the response/recovery time of the sensor at same condition are about 38 and 76 s^35^. C. Balamurugan *et al*.^36^ reported the response time for CrNbO_4_ towards 1000 ppm butane is 6.67 and returns to the base value within 105 s. D.S. Dhawale *et al*.^39^ reported TiO_2_ thin films after irradiation, the response is 1.63 upon exposure of 5200 ppm butane, 130 s and 280 s are corresponding to response and recovery time, respectively. The response of SnO_2_ hierarchical spheres in L.T. Hoa’s article is 8 towards 8000 ppm butane^43^. The low response and slow response and recovery time naturally cannot meet the need of real-time dynamic measurement. But the produced Pd functionalized TiO_2_ nanoparticles only needs 13 and 8 s to response and recovery with 3000 ppm butane with the response of 33.93.

**Table 1:** Comparison of varied material nanostructures in butane sensing performances.

Materials	Concentration (ppm)	Operating temperature (°C)	Response	Ref.
7.5 mol% Pd-TiO_2_	100	340	5.06	This work
	200		7.39	
	500		12.03	
	1000		17.93	
	3000		33.93	
	5000		43.76	
W-TiO_2_	5000	420	22.18	21
Pt-SnO_2_	5000	400	4.6	32
SnO_2_	1000	350	11.76	33
LaFeO_3_	1660	250	14.28	34
MgFe_2_O_4_	2000	425	3.45	35
CrNbO_4_	1000	325	6.67	36
CoFe_2_O_4_	200	250	3.65	37
Pt-ZnO	9000	250	2.44	38
Zn_2_SnO_4_-ZnO	3000		2.70	
TiO_2_	5200	400	1.63	39
Cu_2_ZnSnS_4_	10400	350	2.63	40
In_2_O_3_	1000	135	1.80	41
ZnFe_2_O_4_	500	165	3.85	42
SnO_2_	8000	400	8	43

**Figure 10:**
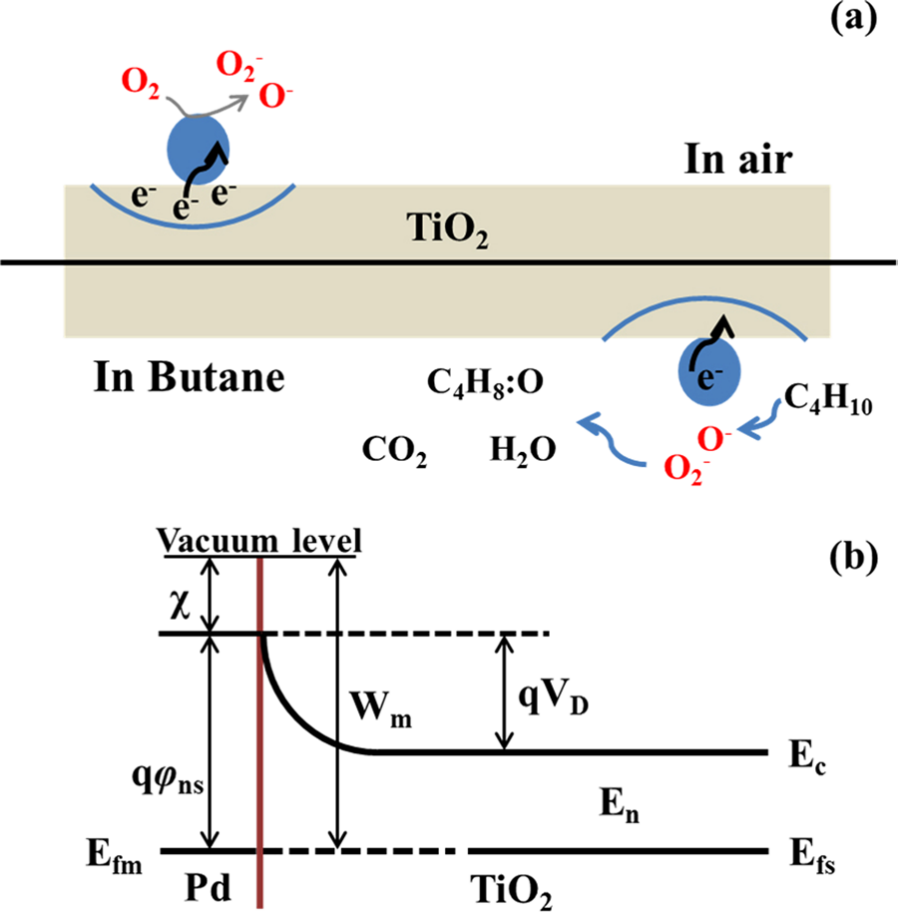
A schematic diagram of the mechanism of PTO-4 nanoparticles based sensor for enhancement caused by metal-semiconductor; Grey represent TiO_2_ nanoparticles; the blue represent palladium nanoparticles.

According to the results of the butane-sensing tests, the Pd functionalized TiO_2_ nanoparticles leads to an unexpected remarkable increase in gas response towards butane. In the previous text we said that ${{\rm{O}}}_{x}^{-}$ have a very important role in the gas sensing property^22^. In generally, the relative amount of absorbed oxygen can be easily detected by XPS by placing the samples in the chamber with high vacuum at room temperature. The conclusion made by X. Liu *et al*.^44^ considered that the relative amount of absorbed oxygen ions changes with different temperatures, especially for ambient air. So, the abundant ambient oxygen resulted by XPS is not suitable for the testing at 340 °C. At 340 °C, electron are excited and captured by oxygen adsorbed on the surface of Pd functionalized TiO_2_ nanoparticles, various kinds of oxygen ions with different valence states are formed, as shown in Fig. 10(a). This leads to electron depletion on the metal oxide surface, which contributes towards the large base line resistance of the sensing material. This process can be described by the following equations^45, 46^:

2 $${{\rm{O}}}_{{\rm{2gas}}}\leftrightarrow {{\rm{O}}}_{{\rm{2ads}}}$$

3 $${{\rm{O}}}_{2{\rm{ads}}}+{{\rm{e}}}^{-}\leftrightarrow {{\rm{O}}}_{2{\rm{ads}}}^{-}$$

4 $${{\rm{O}}}_{2{\rm{ads}}}^{-}+{{\rm{e}}}^{-}\leftrightarrow {{\rm{2O}}}_{{\rm{ads}}}^{-}$$

5 $${{\rm{O}}}_{{\rm{ads}}}^{-}+{{\rm{e}}}^{-}\leftrightarrow {{\rm{O}}}_{{\rm{ads}}}^{2-}$$

When the sensor is exposed to butane, the adsorbed oxygen sites act as active sites for butane gas to get attached. Then butane gas molecules are oxidized by adsorbed oxygen and the electrons are released back to the sensing materials, leading to a thinner space-charge layer^47^. Such process results in a decrease of the resistance and can be expressed by the following equations^21, 39^:

6 $${{({\rm{C}}}_{{\rm{4}}}{{\rm{H}}}_{{\rm{10}}})}_{{\rm{gas}}}\leftrightarrow {({{\rm{C}}}_{{\rm{4}}}{{\rm{H}}}_{{\rm{10}}})}_{{\rm{ads}}}$$

7 $${{\rm{C}}}_{{\rm{4}}}{{\rm{H}}}_{10}+2{{\rm{O}}}^{-}\to {{\rm{C}}}_{{\rm{4}}}{{\rm{H}}}_{{\rm{8}}}{{\rm{O}}}^{-}+{{\rm{H}}}_{{\rm{2}}}{\rm{O}}+{{\rm{e}}}^{-}$$

8 $${{({\rm{C}}}_{{\rm{4}}}{{\rm{H}}}_{{\rm{10}}})}_{{\rm{ads}}}{+\mathrm{13O}}_{{\rm{ads}}}^{-}\to {{\rm{4CO}}}_{{\rm{2}}}{+\mathrm{5H}}_{{\rm{2}}}{{\rm{O}}+\mathrm{13e}}^{-}$$

9 $${{({\rm{C}}}_{{\rm{4}}}{{\rm{H}}}_{{\rm{10}}})}_{{\rm{ads}}}+{{\rm{13O}}}_{{\rm{ads}}}^{2-}\to {{\rm{4CO}}}_{{\rm{2}}}+{{\rm{5H}}}_{{\rm{2}}}{\rm{O}}+{{\rm{26e}}}^{-}$$

While C_4_H_8_O^−^ represents partially oxidized intermediates on the PTO surface. The purpose mechanism of pure TiO_2_ can be proposed by the perspective of absorbed oxygen ions, and the excitation of free electrons only relies on the intrinsic thermal excitation when temperature increases^21^. The results show that the Pd-functionalized TiO_2_ have better performances than the pure one. The enhancement of the sensing performance can be explained as following: when Pd and TiO_2_ get contacted, the metal-semiconductor contact formed^48^, as shown in Fig. 10(b). Work function of Pd and TiO_2_ are 5.12 and 4.2 eV, respectively^49^. Here, *W*_m_ is the work function of Pd, *E*_fm_ and *E*_fs_ are the Fermi level of Pd and TiO_2_, respectively. *E*_c_ is the conduction band level, *E*_n_ is half of the band gap of TiO_2_, the barrier height of Pd is *q*φ_*ns*_ = *W*_m_ − χ, and barrier height of TiO_2_ is *q*V_D_ = *W*_m_ − *W*_s_, here χ is the Fermi level change of TiO_2_ when contacts Pd, *q* is the charge of electrons, *W*_s_ is the work function of TiO_2_. When TiO_2_ and Pd nanoparticles contact with each other, the electrons transfer from TiO_2_ to Pd nanoparticles, and accumulate on the surface of Pd nanoparticles. Based on the equations (2)–(5) and equations (7)–(9), the more oxygen vacancies there are, the more gas molecules can interact. The quantity of atomic oxygen dissociated by Pd diffuses to the TiO_2_ surface. The process is known as the “spillover effect”^19, 20^. Because of the existence of the electron-rich Pd nanoparticles, electron transfer can be realized much more easily. Thus, they provide extra electrons for more oxygen species to adsorb on the surface of the sensing layer, which significantly increases the sensor response. Those results show that Pd functionalized TiO_2_ nanostructures.

## Conclusions

Pd functionalized anatase TiO_2_ nanoparticles were successfully synthesized by a simple, facile hydrothermal method. The morphology, nanostructures and gas sensing properties of the both nanostructures were investigated and compared. The sensor based on PTO-4 exhibits the best performance towards butane, including the preferable response, lower optimum temperature, good response/recovery time, repeatability as well as long-term stability. Meanwhile, the significant enhancement of the response toward butane is attributed to the increased absorbed oxygen ions caused by the formation of metal-semiconductor contact between Pd and TiO_2_ nanoparticles.

## Methods

### Preparation of Pd functionalized TiO_2_ nanoparticles

All the chemical reagents used in the experiments were obtained from commercial sources as guaranteed-grade reagents and used without further purification.

Pd functionalized TiO_2_ nanoparticles were prepared by a simple low temperature hydrothermal method. In a typical synthesized experiment, 4.899 g titanyl sulfate was added to 50 mL deionized water with continual stirring until a homogenous solution was obtained. Then palladium chloride was appended to the aforementioned solution with the molar ratios of 1.0%, 2.5%, 5.0%, 7.5%, and 10.0%, respectively. And they were named as PTO-0, PTO-1, PTO-2, PTO-3, PTO-4 and PTO-5, respectively. Again, the solutions were transferred into the Teflon-lined stainless steel autoclave with a capacity of 80 mL and reacted under hydrothermal conditions at the temperature of 180 °C for 4 h. The autoclaves were cooled down to room temperature in a standard atmosphere. The resulting products were centrifuged, and the precipitates were thoroughly washed with deionized water and dried at 60 °C.

### Characterization of as-synthesized Pd functionalized TiO_2_ nanoparticles

X-Ray diffraction (XRD, Rigaku D/MAX-3B powder diffractometer) with a copper target and K_α1_ radiation (λ = 1.54056 Å) was used for the phase identification, where the diffracted X-ray intensities were recorded as a function of 2θ. The sample was scanned from 20° to 80° (2θ) in steps of 0.02°. Transmission electron microscopy (TEM) measurement was performed on a Zeiss EM 912 Ω instrument at an acceleration voltage of 120 kV, while high-resolution transmission electron microscopy (HRTEM) characterization was done using JEOL JEM-2100 Electron Microscope (with an acceleration voltage of 200 kV). The samples for TEM were prepared by dispersing the final dry samples in ethanol, and this dispersing was then dropped on carbon-copper grids covered by an amorphous carbon film. The selected area electron diffraction (SAED) and energy-dispersive X-ray spectroscopy (EDX) spots pattern scanning analysis was performed by the TEM attachment. The nitrogen adsorption isotherm was measured at 77.3 K with a Micromeritics ASAP 2010 automated sorption analyzer. Prior to the measurement, the sample was degassed at 300 °C for 6 h under a vacuum. X-ray photoelectron spectroscopy (XPS) was carried out at room temperature in an ESCALAB 250 system. During XPS analysis, an Al K_α_ X-ray beam was adopted as the excitation source and the vacuum pressure of the instrument chamber was 1 × 10^−7^ Pa as read on the panel. Measured spectra were decomposed into Gaussian components by a least-square fitting method. Bonding energy was calibrated with reference to the C1s peak (284.6 eV).

### Preparation and test of gas sensor

The fabrication of indirect-heating structure sensor was described in the literature^50^. Pd functionalized TiO_2_ nanoparticles were mixed with deionized water to form pastes, and then coated onto the outside of an alumina tube with a pair of Au electrodes and platinum wires installed at each end. A Ni-Cr alloy wire crossing the alumina tube was used as a resistor to ensure both substrate heating and temperature control by adjusting the heating voltage (*V*_h_). Before measuring the gas sensing properties, the gas sensors were aged at a voltage of 5 V to improve their stability and repeatability. Gas sensing properties were measured by a WS-30A system (Weisheng Instruments Co. Zhengzhou, China) covered with a chamber (18 L in volume). During the test, the desired amounts of test gas were injected into a test chamber using a microinjector after the base line of the sensor was stable. The desired concentrations of the testing gas are obtained by the volume of the analyte solution. An evaporator and two fans are installed to make the gas homogeneous immediately in the chamber. Note that the clean dry air was used as a reference gas and diluting gas for the different concentrations of target gas. It is well known that the sensor response value (*β*) was defined as the ratio of the electrical resistance in air (*R*_a_) to that in target gas (*R*_g_), namely *β* = *R*_a_/*R*_g_^51^ for n-type gas sensors. Meanwhile, the operating temperature of the sensors was varied in the range from 260 to 485 °C.
